# Thalamic reticular nucleus in the pathophysiology of schizophrenia

**DOI:** 10.4103/NRR.NRR-D-25-00928

**Published:** 2026-01-27

**Authors:** Gonzalo Flores, David J. Apam-Castillejos, Hannaford Edwards, Victor M. Magdaleno-Madrigal, Juan Nacher, Hiram Tendilla-Beltrán, Lalit K. Srivastava

**Affiliations:** 1 Instituto de Fisiología, Benemérita Universidad Autónoma de Puebla (BUAP), Puebla, Mexico; 2 Douglas Hospital Research Centre, Department of Psychiatry, McGill University, Montreal, QC, Canada; 3 Instituto Nacional de Psiquiatría Ramón de la Fuente Muñiz, Mexico City, Mexico; 4 Neurobiology Unit, Institute for Biotechnology and Biomedicine (BIOTECMED), Universitat de Valencia, Valencia, Spain; 5 Spanish National Network for Research in Mental Health, CIBERSAM, Madrid, Spain; 6 Fundación Investigación Hospital Clínico de Valencia, INCLIVA, Valencia, Spain; 7 Centro de Investigación en Reproducción Animal, Cinvestav-UAT, Tlaxcala, Mexico

**Keywords:** animal model, corticolimbic system, dendritic spines, gamma-aminobutyric acid, glutamate, mushroom spines, parvalbumin-positive cells, prefrontal cortex, psychosis, pyramidal neurons, thalamo-cortical loop

## Abstract

Mechanistic analyses on schizophrenia have traditionally focused on corticolimbic structures such as the prefrontal cortex, hippocampus, and amygdala, given their established roles in cognition. However, the thalamus, a critical hub that interconnects these regions, has garnered comparatively less attention. Of particular interest is the thalamic reticular nucleus, which plays a crucial role in cognition and sensory processing through its connections with the dorsomedial thalamic nucleus and the prefrontal cortex. A potential role of the thalamic reticular nucleus in schizophrenia has been suggested in a few reports; however, recent analyses have identified a specific link between the thalamic reticular nucleus and layer 5 neurons of the prefrontal cortex, a relationship highlighted by our group’s earlier findings from 2012, which demonstrated that bilateral thalamic reticular nucleus lesions in adult rats caused neuronal atrophy in these cortical neurons. Building on this foundation, this manuscript explores the role of the thalamic reticular nucleus in schizophrenia neurobiology by reviewing its functional neuroanatomy, its integration within the corticolimbic system, and mechanisms of its potential involvement in the disease. This hypothesis is further developed by describing our novel findings from rats with neonatal ventral hippocampus lesion, widely considered a developmental model for schizophrenia. For the first time, we report dendritic spine pathology in thalamic reticular nucleus neurons, characterized by reduced spine density and a lower proportion of mushroom spines. Furthermore, we demonstrate a decrease in the density of parvalbumin-positive cells within the thalamic reticular nucleus in the neonatal ventral hippocampus lesion model. Together, these findings suggest significant alterations in the corticolimbic network and position the thalamic reticular nucleus as a complex yet promising mechanistic contributor to the neurobiology of schizophrenia.

## Neurobiology of Schizophrenia

The neurobiology of schizophrenia, despite being recognized as a distinct condition since the late 1800s, remains poorly understood (Cummings et al., 2024). This gap in understanding is highlighted by the recent U.S. Food and Drug Administration approval (September 2024) of a new treatment for schizophrenia in over 60 years, even though the disorder affects nearly 1% of the global population (Anderer, 2024). Despite extensive research, schizophrenia continues to pose significant challenges in fully deciphering its underlying pathophysiology.

This debilitating condition not only impacts individuals but also places a heavy burden on their families. In recent years, significant progress has been made in understanding its pathophysiology, driven by advances in brain morphology analysis — particularly in examining connectivity between brain regions — and genome-wide association studies (Consortium, 2014; Howes et al., 2023). These studies have underscored the critical roles of impaired neurotransmission and synaptic plasticity as central mechanisms of the disease (Trubetskoy et al., 2022). Interestingly, much of this emerging evidence converges on the neurodevelopmental hypothesis of schizophrenia, proposed by Weinberger (1987) and expanded by Murray and Lewis (1987). According to this hypothesis, disruptions during early brain development can impair connectivity between cortical and subcortical regions, laying the groundwork for the emergence of schizophrenia during adolescence or early adulthood. Building on this framework, Keshavan et al. (1994) refined the hypothesis by incorporating the concept of excessive synaptic pruning, an idea first introduced by Feinberg (1982). Post-mortem analyses revealing reduced dendritic spine density in the neocortex of individuals with schizophrenia support this theory. Glantz and Lewis (2000) proposed that early developmental insults may lead to excessive pruning of synapses, thereby compromising neural function and increasing the risk of developing schizophrenia. Importantly, the role of genetic factors in predisposing individuals to abnormal neurodevelopment has become increasingly evident. Recent findings have been integrated into the neurodevelopmental hypothesis, highlighting multiple candidate genes involved in synaptic function and innate immunity. This has led to the formulation of the developmental risk factor model for schizophrenia, as proposed by Murray et al. (2022).

## Search Strategy

We conducted a comprehensive literature search of articles published between 1982 and the present using PubMed and Google Scholar. The search employed the following keywords and combinations thereof: “neurodevelopmental hypothesis of schizophrenia,” “dendritic spines in schizophrenia,” “thalamic reticular nucleus in schizophrenia,” “thalamic reticular nucleus in psychiatric disorders,” “oxidative stress in schizophrenia and depression,” and “parvalbumin neurons in schizophrenia.” In this narrative review, we summarize evidence supporting the neurodevelopmental hypothesis of schizophrenia, with a focus on the disruption of the thalamo-cortical loop as a core neurobiological feature of the disease. This is further supported by unpublished data on neuroplasticity and biochemical alterations in the thalamic reticular nucleus, obtained from a developmental animal model relevant to the study of schizophrenia.

## Schizophrenia as a Neurodevelopmental Disorder

**Figure 1:**
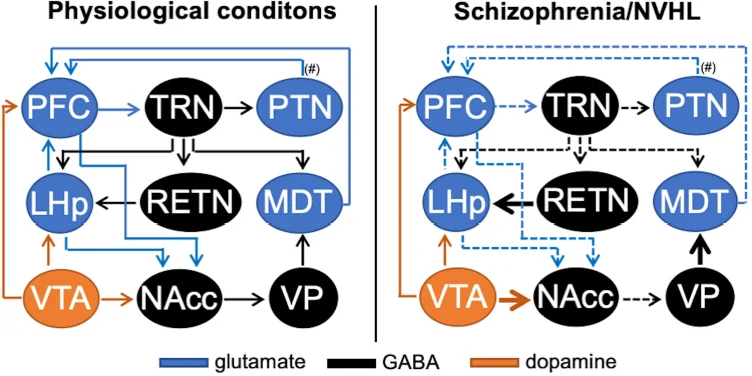
Role of the TRN in schizophrenia. Corticolimbic circuit interactions with thalamic nuclei under physiological conditions, in schizophrenia, and in rats with NVHL. For details, see the text. #Projections from the PTN to the PFC have only been described in primates (Leow et al., 2022). In rodents, the homologous region is the lateral posterior thalamus, which projects to the PFC. Dotted lines indicate impaired connectivity, and thicker lines indicate increased connectivity. For further details, refer to the main text. GABA: Gamma-aminobutyric acid; LHp: limbic hippocampus (anterior in primates, ventral in rodents); MDT: medial-dorsal thalamic nucleus; NAcc: nucleus accumbens; NVHL: neonatal ventral hippocampal lesions; PFC: prefrontal cortex; PTN: pulvinar thalamic nucleus; RETN: reuniens nucleus of the thalamus; TRN: thalamic reticular nucleus; VP: ventral pallidum; VTA: ventral tegmental area.

In humans, the development of the brain begins from the ectoderm; at about 21 days of gestation, neural crests emerge on the back of the embryo. These crests grow to form the neural tube by the end of the first month, which then undergoes division into three vesicles. Later, between the third and fourth months of gestation, the cortices, including archicortices and neocortical regions, start to develop (Stiles and Jernigan, 2010; Vaid and Huttner, 2022). The prefrontal cortex (PFC), a neocortical structure, comprises six layers and plays a crucial role in cognitive function. During the third and fourth months of gestation, these layers establish internal connections and later connect with other cortices. Connections between the thalamus and cortices begin to form during the fifth month. In the context of schizophrenia, connections involving the limbic thalamus, especially those between the thalamic mediodorsal nucleus (MDT) and the PFC, are significant (Phillips et al., 2021; Leow et al., 2022; **Figure 1**). From the seventh month of intrauterine life, connections between the hippocampus and amygdala with various cortical regions emerge (Gabard-Durnam et al., 2018). The CA1 subregion of the limbic hippocampus (anterior in primates and ventral in rodents) and the basolateral amygdala form connections with the PFC and the nucleus accumbens (NAcc), known as the reward nucleus (Kolk and Rakic, 2022). Additionally, the PFC sends glutamatergic projections to GABAergic inhibitory neurons in structures such as the thalamic reticular nucleus (TRN) and the NAcc (**Figure 1**). At birth, the human brain already possesses essential connections for proper functioning, unlike rodents, whose brains are born in a less mature state regarding inter-regional connections (Zikopoulos and Barbas, 2006; Carroll et al., 2022; Hádinger et al., 2023).

The development of neural connections follows a specific sequence, and insults during this process can impact both the quantity and quality of connections within limbic regions. In schizophrenia, it is suggested that risk factors or early insults disrupt the development of connections between the PFC and the thalamus, hippocampus, and basolateral amygdala (**Figure 1**). These connections typically begin during the second trimester of pregnancy, and supporting evidence comes from imaging studies showing macro and microstructural changes in these brain regions in individuals with schizophrenia (Murray et al., 2022; Reyes-Lizaola et al., 2024). Genetic factors can disrupt the expression of key signaling molecules during development, such as neurotransmitter receptors, adhesion molecules, and complement-related proteins involved in synapse pruning, compromising connectivity between brain regions linked to schizophrenia (Gulsuner et al., 2020; Merikangas et al., 2022; Hoffman et al., 2023). However, these disrupted connections are not solely driven by genetics; environmental factors, including maternal stress, caregiving, social interactions, and learning experiences, may also play a critical role.

Among the brain regions implicated in schizophrenia, the corticolimbic system has been extensively studied due to its involvement in reality perception, interpretation, and the regulation of behavioral responses (Sokolowski and Corbin, 2012). Reports highlight the collaborative regulation of PFC functioning by the hippocampus and basolateral amygdala, collectively contributing to proper NAcc functioning. Interestingly, the environment is proposed to be a critical factor in PFC functionality, consequentially impacting NAcc functionality (Knight et al., 2022; **Figure 1**). A highly stressful environment likely impairs cortical function, particularly affecting the connectivity between the prefrontal and temporolimbic cortices (Selemon and Zecevic, 2015; Li et al., 2025). Additionally, elevated stress levels during developmental phases and maternal infection are potential contributors, as suggested by animal models (Martínez-Téllez et al., 2009; Bergdolt and Dunaevsky, 2019).

## Thalamic Reticular Nucleus in the Schizophrenia Neurobiology

Thus, substantial information on the dysfunctional corticolimbic structures at genetic, molecular, functional, and structural levels has been documented for schizophrenia pathophysiology (Tendilla-Beltrán et al., 2021; Howes et al., 2024). However, multiple studies have suggested that the etiological basis of corticolimbic impairment in schizophrenia could also be linked to abnormalities in thalamic function (Clinton and Meador-Woodruff, 2004; Pergola et al., 2015). Among the various thalamic nuclei implicated in the pathophysiology of schizophrenia, including the MDT, a recent study has identified dysfunction of the TRN as a potential neurobiological mechanism underlying the disorder (Steullet et al., 2018). This is because the TRN plays a crucial role in inhibiting the activity of thalamic nuclei, particularly those associated with limbic functions, such as the MDT. Additionally, it receives glutamatergic projections from hypofunctional layer 5 of the PFC in individuals with schizophrenia (Mease and Gonzalez, 2021; **Figure 1**).

The TRN, a thin shell-like structure that encases the lateral thalamus, is bordered by the external medullary lamina and extends almost the full depth of the thalamus (Pinault, 2004). In general, the TRN has connections with most thalamic nuclei (Crabtree, 2018). However, first- and second-order pathways have been identified based on their connections with cortical and subcortical structures involved in various tasks, such as sensory perception and cognition (Li et al., 2020). Its strategic location within the brain plays a crucial role in the cortico-thalamo-cortical circuit, facilitating waxing-and-waning oscillatory activity in the 12–16 Hz range in the hippocampus and neocortex. This oscillatory activity has been associated with memory consolidation (Mölle and Born, 2011). At the cellular level, the TRN is composed of GABAergic inhibitory neurons characterized by the expression of glutamate decarboxylase 67 and parvalbumin (PV), which is expressed in approximately 70% of these cells (Klimczak et al., 2024). These neurons exhibit distinct properties, including a specific topographic distribution. The TRN is primarily composed of two types of cells: core (inner neurons) and edge neurons, which have distinct structural and functional characteristics (Lam and Sherman, 2011; Li et al., 2020; Martinez-Garcia et al., 2020). Core neurons are located mainly in the central region of the TRN and are distinguished by their larger somas and more extensive dendritic trees, allowing for greater integration of synaptic inputs. Additionally, these cells almost exclusively express secreted phosphoprotein 1 gene (Li et al., 2020). These neurons predominantly receive inputs from first-order thalamic relay nuclei, such as the ventroposterior medial nucleus, and play a crucial role by providing potent inhibitory feedback to thalamic relay cells, thereby regulating the flow of sensory information to the cortex. Their high-frequency burst activity (**~**300 Hz compared to **~**180 Hz of edge neurons) following hyperpolarization is vital for synchronizing thalamocortical oscillations, and ultimately for attention and memory (Martinez-Garcia et al., 2020; Vaughn et al., 2024). In both core and edge neurons, burst firing in both subtypes relies on low-threshold T-type calcium currents, with core neurons showing a higher propensity for bursts due to enhanced calcium influx (Li et al., 2020; Klocke et al., 2024).

In contrast, edge neurons, located at the periphery of the TRN, are characterized by smaller somas and simpler dendritic arbors, which result in reduced synaptic integration. These neurons also selectively express the endothelin-converting enzyme-like 1 gene (Li et al., 2020). These neurons primarily receive inputs from higher-order thalamic nuclei, such as the posterior medial nucleus. Consequently, edge neurons contribute to attentional control and higher-order sensory processing through less synchronous but more selective inhibition, despite having similar inhibitory properties. The intrinsic characteristics of edge neurons, including a higher membrane resistance (**~**220 MΩ compared to **~**120 MΩ of core neurons) and a slightly less negative resting membrane potential (**~**66 mV compared to **~**70 mV of core neurons), result in distinct firing patterns. While these properties reduce the likelihood of burst firing, they also suggest that edge neurons may exhibit greater excitability. These differences enable core and edge neurons to make unique contributions to thalamocortical communication and sensory information processing (Martinez-Garcia et al., 2020; Carroll et al., 2022; Vaughn et al., 2024). Additionally, the role of the TRN in cognitive functions such as attention regulation has been described since the 1980s. Cases of domoic acid poisoning from mussel consumption have demonstrated impaired attention processes, with imaging analyses revealing damage to the TRN in affected individuals (Todd, 1993). This was later confirmed by the direct injection of domoic acid into the TRN in the brains of rodents (Friedberg and Ross, 1993; Guillery et al., 1998).

Given its role in sensory perception and cognitive functions, the TRN has been investigated as a neurobiological substrate in the pathophysiology of various psychiatric disorders, particularly those characterized by psychosis, a loss of contact with reality, as seen in schizophrenia (Steullet et al., 2018; Gerardo and Manuel, 2020). In this disorder, the TRN shows reduced PV neuron density and a loss of associated perineuronal nets (PNNs), which are crucial for antioxidant defense in these cells. Consequently, oxidative stress-related neuronal damage has also been observed in postmortem samples (these mechanisms will be discussed in detail later) (Steullet et al., 2018). Although the TRN has not been specifically studied with imaging techniques in patients, mainly because of its small size and elongated shape, a meta-analysis of over 18,000 individuals found a reduced thalamic volume in schizophrenia (Haijma et al., 2013). Interestingly, this reduction was observed even in the pre-psychosis phase, supporting the neurodevelopmental hypothesis of the disease, yet it can also be influenced by antipsychotic exposure, which may involve non-monoaminergic drug mechanisms (Tendilla-Beltrán et al., 2021). Additionally, schizophrenia is associated with disrupted thalamo-cortical inhibitory gating and altered thalamic connectivity patterns that correlate with cognitive and sensory symptoms. Collectively, these abnormalities position the TRN as a central site of pathology, potentially underlying the sensory, cognitive, and sleep disturbances characteristic of the disorder (Anticevic and Halassa, 2023).

## Neonatal Ventral Hippocampus Lesion Rat Model and the Role of the Thalamic Reticular Nucleus in the Neuronal and Behavioral Phenotypes Relevant to Schizophrenia

**Figure 2:**
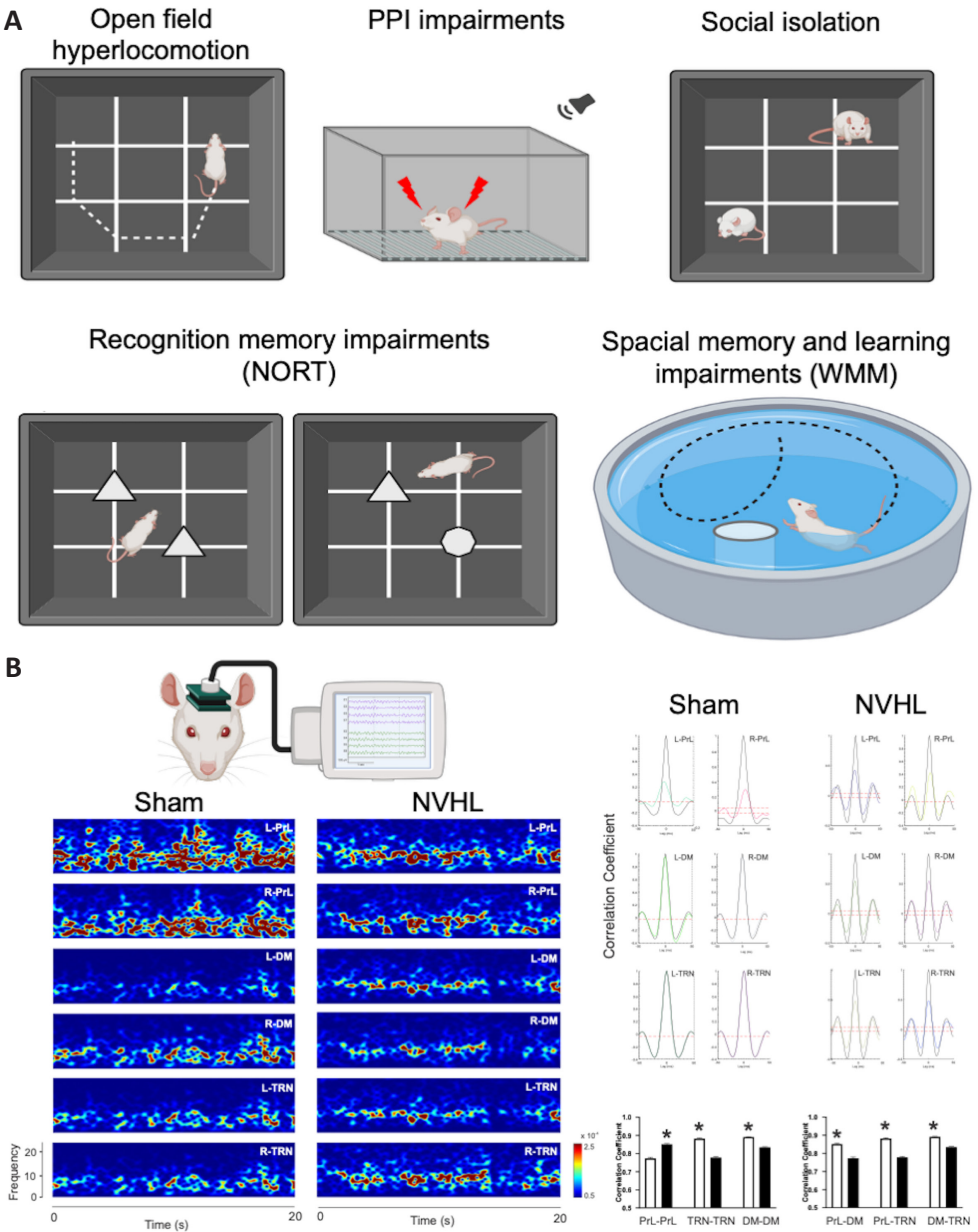
Behavioral and LFP alterations in rats with NVHL. (A) Behavioral alterations in rats with NVHL. A myriad of behavioral alterations has been described in the NVHL model, including hyperlocomotion in open-field, impaired prepulse inhibition evidenced by increased startle response, social isolation, impaired recognition memory evidenced by the poor performance in the NORT, impaired special memory and learning evaluated with the WMM. (B) LFPs in rats with NVHL (unpublished data). Representative spectrograms of LFPs and coherence in 0.5–25 Hz bandwidths. Images with rats were created with BioRender.com. DM: Dorsomedial thalamic nucleus; L: left; LFP: local field potential; NORT: novel object recognition test; NVHL: neonatal ventral hippocampus lesion; PrL: prelimbic cortex (subregion of the prefrontal cortex); R: right; TRN: thalamic reticular nucleus; WMM: water Morris maze.

In animal models used to study schizophrenia, such as neonatal ventral hippocampus-lesioned (NVHL) rats, behavioral changes emerge after puberty due to early disconnection between limbic regions (Tseng et al., 2009; **Figure 2A**), which can be attenuated with antipsychotic treatment (Tendilla-Beltrán et al., 2021). Similar to findings in postmortem samples from patients with schizophrenia (Glausier and Lewis, 2013), NVHL animals exhibit dendritic spine pathology in the pyramidal neurons of the PFC (Tendilla-Beltrán et al., 2019). Further support for the TRN in schizophrenia-related mechanisms comes from our finding that short-term deep brain stimulation of TRN restored the impaired cortical electroencephalographic (EEG) activity in animals with NVHL (Magdaleno-Madrigal et al., 2017; **Figure 2B**). Interestingly, our group demonstrated that excitotoxic lesions in the TRN lead to a reduction in dendritic spines and dendritic arborization in layers 3 and 5 of the PFC (Torres-García et al., 2012), a finding that aligns very well with the post-mortem literature in schizophrenia. To our knowledge, this was the first report suggesting the connection between the TRN and the PFC, which was later described in detail with neurophysiological techniques (Carroll et al., 2022; Hádinger et al., 2023).

The organization of corticothalamic pathways in mice is specifically regulated through the layer 5 neurons, highlighting the specialized cortical regulation of thalamic function and its essential role in frontal cortex function (Carroll et al., 2022; Hádinger et al., 2023). Interestingly, the stimulation of the TRN improves reduced social interaction in the NVHL animals, likely due to correcting deficits in PFC function (Magdaleno-Madrigal et al., 2017). Additionally, rats with NVHL have a reduction in mushroom dendritic spines, structures associated with synaptic strengthening, in multiple corticolimbic areas, including the PFC, NAcc, and basolateral amygdala (Tendilla-Beltrán et al., 2019). This reduction, together with the overall decrease in dendritic spine density, constitutes what our working group has recently termed “dendritic spine degeneration” (Flores et al., 2025). TRN lesions in adult animals not only impair the connections with the PFC but also impair the connectivity within the CA1 region of the ventral hippocampus and the NAcc — regions implicated in the pathophysiology of schizophrenia (Torres-García et al., 2012). This information underscores the significance of the TRN in models utilized in understanding schizophrenia.

While there is limited information about the role of structural neuroplasticity in TRN function, analyses in other regions, such as the PFC, NAcc, and amygdala, have demonstrated its relationship to the schizophrenia-related behavioral phenotype in animal models (Tendilla-Beltrán et al., 2021). In 1966, Scheibel and Scheibel performed a fundamental work describing the neural morphology of TRN neurons in mice, rabbits, and cats with the Golgi technique. In this manuscript, we present unpublished data on the effects of the NVHL in rats on the structural neuroplasticity of core TRN neurons, using the Golgi-Cox whole brain staining method (Gibb and Kolb, 1998).

## Overview of Unpublished Experimental Findings on Rats with Neonatal Ventral Hippocampus-lesioned and Thalamic Reticular Nucleus Histology

Pregnant Sprague-Dawley rats at gestational days 14–16 were obtained from the facilities of the Autonomous University of Puebla and Charles River Canada. The animals were housed individually in a temperature- and humidity-controlled environment with a 12-hour light/dark cycle and had free access to food and water. The day of birth was recorded as postnatal day 0 (P0). On postnatal day 7 (P7), only male pups (*n* = 24) were randomly assigned to either a sham group (*n* = 12) or a NVHL group (*n* = 12). All experimental procedures were conducted in accordance with the BUAP Animal Care Committee (FLAG-UALVIEP-17-1), the Guide for the Care and Use of Laboratory Animals of the Mexican Council for Animal Care (Norma Oficial Mexicana NOM-062-ZOO-1999), and the Canadian Council on Animal Care. Ethical approval was also obtained from the McGill University Animal Care Committee. Every effort was made to minimize the number of animals used and to reduce animal suffering. The NVHL procedure was carried out according to the protocol established by Flores et al. (1996). On postnatal day 7 (P7), male pups (weighing 15–18 g) were anesthetized via hypothermia and positioned on a custom platform designed for neonatal rats (Sierra et al., 2009), which was secured to a Kopf stereotaxic instrument. A total volume of 0.3 µL of ibotenic acid (10 µg/µL; Sigma-Aldrich, USA) or vehicle (0.1 M phosphate-buffered saline, pH 7.4) was bilaterally injected into the ventral hippocampus over a 2-minute period using a 30-gauge stainless steel cannula connected to a Hamilton syringe and an infusion pump. The injection coordinates, based on previous reports (Lipska et al., 1993; Flores et al., 1996; Tendilla-Beltrán et al., 2019), were: anterior-posterior −3.0 mm, medial-lateral ±3.5 mm from bregma, and dorsal-ventral −5.0 mm from the dura. Following surgery, pups were placed on a heating pad to recover before being returned to their dams.

On postnatal day 60 (P60), adult rats were deeply anesthetized with sodium pentobarbital (75 mg/kg, intraperitoneally) and euthanized in accordance with the approved experimental protocols. Two groups of animals were used for different analyses. In the Golgi–Cox staining group (*n* = 8 per group), rats were perfused intracardially with physiological saline, after which their brains were removed and immersed in Golgi–Cox solution for histological processing. In the immunohistochemistry group (*n* = 4 per group), rats were perfused first with physiological saline, followed by 4% paraformaldehyde in 0.1 M phosphate-buffered saline. Their brains were then extracted, post-fixed in 4% paraformaldehyde for 24 hours, cryoprotected, and frozen until further use.

To assess dendritic spine plasticity in the TRN following NVHL, the Golgi–Cox staining technique was used, following the protocol by Gibb and Kolb (1998) with modifications from Flores et al. (2005). Coronal brain sections (200 µm thick) at the level of the PFC were obtained using a vibratome (MA752; Campden Instruments, England) and mounted onto clean, gelatin-coated microscope slides. The slides were treated with ammonium hydroxide for 30 minutes in the dark, followed by 30 minutes in Kodak Rapid Film Fixer, also in the dark. Afterward, they were rinsed with distilled water and dehydrated through a graded ethanol series (50%, 75%, and 90% for 1 minute each, followed by 100% ethanol twice for 5 minutes each). The sections were then cleared in xylene for 15 minutes and coverslipped using a synthetic resin mounting medium.

**Figure 3:**
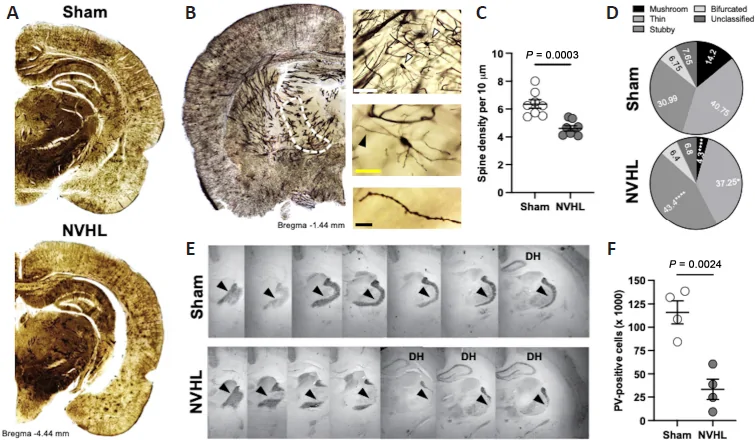
Neuroplasticity alterations in the TRN in rats with NVHL. (A) Verification of the lesion. Golgi-Cox staining was performed on coronal brain sections from rats subjected to either a sham lesion or NVHL. Loss of ventral hippocampal integrity was observed only in NVHL rats. (B) Panoramic coronal section of the rat brain stained with Golgi-Cox showing the TRN (white line) and a characteristic pair of neurons of this nucleus (white arrowheads). Additionally, an isolated TRN neuron with a dendrite (black arrowhead) is shown in which dendritic spine density and the morphological spine classification were assessed. (C, D) Structural neuroplasticity parameters (unpublished data). (C) Dendritic spine density. The NVHL reduced the dendritic spine density in TRN neurons (Student’s *t* test: *t*_(14)_ = 4.818). (D) Dendritic spine morphological classification. The NVHL caused a reduction in the proportion of both mushroom (two-way analysis of variance, lesion × type of spine *F*_(4, 70)_ = 46.66, *P* < 0.0001; Sidak’s multiple comparisons test: ****P < 0.0001) and thin spines (Sidak’s multiple comparisons test: *P = 0.0168). However, the NVHL increased the proportion of stubby spines (Sidak’s multiple comparisons test: ****P < 0.0001). (E) Detection of PV-positive cells in the TRN using immunohistochemistry (arrowheads). (F) Quantification of PV-positive cells (unpublished data). The NVHL reduced the PV-positive cell density (Student’s *t* test: *t*_(6)_ = 5.01). Stereotaxic references are from Paxinos and Watson (1998). Data presented in this figure is unpublished. DH: Dorsal hippocampus; LHp: limbic hippocampus (anterior in primates, ventral in rodents); MDT: medial-dorsal thalamic nucleus; NAcc: nucleus accumbens; NVHL: neonatal ventral hippocampus lesion; PV: parvalbumin; RETN: reuniens nucleus of the thalamus; TRN: thalamic reticular nucleus; VP: ventral pallidum; VTA: ventral tegmental area.

Then, the verification of the ventral hippocampus lesion was assessed as previously described (Tendilla-Beltrán et al., 2024). In brief, coronal sections stained with Golgi-Cox were examined at the level of the ventral hippocampus (bregma –4.44 to –5.8 mm) using an optical microscope. In both strains, rats with NVHL exhibited clear morphological disruption of the ventral hippocampus (**Figure 3A**), although some adjacent regions remained intact. In contrast, rats with sham lesions showed no signs of damage.

Dendritic arbor segments of TRN neurons were identified using coordinates from the rat brain atlas (Paxinos, 1998; **Figure 3B**). For each animal, five dendritic segments (with a minimum length of 10 µm) were selected from each hemisphere and reconstructed using a camera lucida at 1000× magnification on a DMLS Leica microscope to estimate dendritic spine density, following the procedures described by Flores et al. (2005) (**Figure 3B**). Spine morphology was evaluated on the same segments using a 2× lens at 2000× magnification. Fifty consecutive spines per segment were analyzed and classified according to the shape of their head and neck, based on criteria adapted from Tendilla-Beltrán et al. (2019). Spines were categorized into six types: mushroom (prominent head with a well-defined neck), thin (elongated with similar thickness in head and neck), stubby (broad with an indistinct neck), bifurcated (two-headed), multi-headed (more than two heads), and unclassified (not fitting any of the previous categories). All measurements and classifications were performed by a trained observer who was blinded to the experimental conditions.

Immunohistochemistry was assessed for PV as previously reported (Muller et al., 2006). For this purpose, 40 µm-thick coronal sections containing the TRN were collected using a cryostat. Free-floating sections were processed using the avidin-biotin peroxidase method, with 3,3′-diaminobenzidine (DAB) as the chromogen. Following an initial rinse in phosphate buffer, endogenous peroxidase activity was blocked by incubating the sections in a solution of 3% hydrogen peroxide and 10% methanol in phosphate buffer at room temperature. The sections were then rinsed again in phosphate buffer and washed in Tris-buffered saline containing 0.5% Triton X-100 (TBS-TX). Blocking was performed using 5% normal goat serum in TBS-TX for 1 hour at room temperature. Sections were then incubated overnight at 4°C with a monoclonal mouse anti-PV primary antibody (1:4000 in TBS-TX). The following day, after rinsing, sections were incubated overnight at 4°C with a biotinylated goat anti-mouse secondary antibody. After additional rinses, sections were incubated for 1.5 hours with the VectaStain ABC HRP kit, according to the manufacturer’s instructions. They were subsequently rinsed in TBS-TX and Tris-HCl buffer before being developed in DAB. The DAB solution was prepared by dissolving a 10 mg DAB tablet in 20 mL of Tris-HCl buffer, heated and stirred for 2 hours. Hydrogen peroxide was then added, and the solution was cooled in the freezer for 20 minutes to slow the enzymatic reaction. Sections were incubated in the DAB solution for approximately 3 minutes, rinsed in Tris-HCl, mounted on slides, and coverslipped using Entellan (Merck Millipore, Darmstadt, Germany).

For PV cell count, 1 in 6 series brain slices were analyzed using an optical microscope and sampled at 20× in a 250 × 250 µm area. Images were then analyzed with the ImageJ software (NIH, USA), and only well-stained and complete neurons were quantified.

## NVHL Induces Dendritic Spine Plasticity Deficits and Parvalbumin Cell Loss at Adulthood

At adulthood (60 postnatal days), the NVHL led to a reduction in dendritic spine density in TRN neurons (Student’s *t* test: *t*_(14)_ = 4.818, *P* = 0.0003; **Figure 3C**), primarily due to a decrease in the proportion of mushroom spines (two-way analysis of variance: lesion × type of spine *F*_(4, 70)_ = 46.66, *P* < 0.0001; Sidak’s multiple comparisons test: *P* < 0.0001) and secondarily due to the loss of thin spines (Sidak’s multiple comparisons test: *P* = 0.0168; **Figure 3D**). These results establish dendritic spine degeneration in the TRN in the NVHL model, as previously described in corticolimbic regions (Tendilla-Beltrán et al., 2019; Flores et al., 2025). Mushroom spines are considered fully functional, synapse-related structures that contribute to the maintenance of neuronal circuits. Conversely, thin spines are associated with synaptogenesis and likely play a crucial role in the structural and functional reorganization of neuronal circuits (Hering and Sheng, 2001). Interestingly, the NVHL also increased the proportion of stubby spines (Sidak’s multiple comparisons test: *P* < 0.0001; **Figure 3D**), which are involved in calcium dynamics at the spine-dendrite interface but contribute minimally to synaptic transmission (Majewska et al., 2000; Bell et al., 2022; Rosado et al., 2022). This reduction in dendritic spine density, accompanied by the loss of mushroom spines, is consistent with dendritic spine pathology (Glausier and Lewis, 2013) observed in various corticolimbic regions of NVHL-affected rats (Tendilla-Beltrán et al., 2019). Additionally, the number of PV-positive cells was detected by immunoreactivity and quantified in the TRN (**Figure 3E**), as previously reported for the PFC (Cabungcal et al., 2014). The NVHL drastically decreased the number of PV-positive cells in the TRN in comparison with the sham group (Student’s *t* test: *t*_(6)_ = 5.01, *P* = 0.0024; **Figure 3F**). Thus, our data support the potential role of TRN neuron dendritic spine pathology and PV-positive cell loss, along with corticolimbic dysfunction, in the behavioral alterations observed in NVHL animals, such as increased locomotion in novel environments (Lipska et al., 1993), altered social behavior (Sams-Dodd et al., 1997; Silva-Gómez et al., 2003), and impaired recognition memory (Tendilla-Beltrán et al., 2019) (**Figure 2A**). However, a critical question is, what role does the TRN play in this impaired corticolimbic circuitry in the NVHL and schizophrenia (**Figure 1**)?

## Contribution of Thalamic Reticular Nucleus Alterations to Schizophrenia-related Phenotypes in the Neonatal Ventral Hippocampus-lesioned Model

Limbic thalamic nuclei are complex; however, the primary limbic nucleus is the MDT. Comprising glutamatergic neurons with spontaneous activity, the MDT sends excitatory projections to limbic cortical regions. Neurons in the MDT are under the regulatory influence of inhibitory inputs from the TRN and ventral pallidum (VP). They both receive excitatory glutamatergic inputs from the CA1 region of the hippocampus, the basolateral amygdala, and the PFC (Kolk and Rakic, 2022; Pinault, 2023; **Figure 1**). Under resting conditions, inhibitory inputs from the TRN and VP serve to inhibit the activity of glutamatergic neurons in the MDT of the thalamus. This inhibition results from the spontaneous activity of GABAergic neurons in the TRN and VP. However, when the PFC, hippocampus, and amygdala become activated, the excitatory inputs to the MDT come into play. These excitatory inputs also activate the medium spiny neurons of the NAcc, which are GABAergic. As a result, the GABAergic neurons of the VP nucleus are inhibited, leading to the disinhibition of MDT nucleus neurons (**Figure 1**). Consequently, the inhibitory effect of the TRN, regulated by the excitatory input from the PFC, serves as a brake on the increase in excitatory inputs to the MDT. In schizophrenia, the hypo-functionality of the PFC, particularly in layer 5, may result in reduced stimulation of the TRN. This, in turn, will weaken the inhibitory control on the MDT. In this way, the loss of postsynaptic glutamatergic surface, resulting from reduced dendritic spine density and a decreased population of mushroom spines, may disrupt the excitation/inhibition balance in the TRN, as occurs in pyramidal neurons (Iascone et al., 2020) and striatum’s’ medium spiny neurons, also known as spiny projection neurons (Garcia et al., 2010). Consequently, its inhibitory influence on limbic structures such as the hippocampus may be impaired (**Figure 1**), leading to thalamocortical oscillation disturbances and, ultimately, cognitive deficits, as widely documented in both patients and rats with NVHL (Latchoumane et al., 2017; Tendilla-Beltrán et al., 2021; McCutcheon et al., 2023). Given the crucial role of the TRN, this could explain the decrease in attentional processes observed in schizophrenia and could have implications for social interaction. This idea is supported by our previous published study where the stimulation of the TRN in the NVHL model led to an increase in social interaction (Magdaleno-Madrigal et al., 2017).

## Thalamic Reticular Nucleus Alterations, a Common Feature in Animal Models for the Study of Schizophrenia

Additionally, in other animal models relevant to schizophrenia, it has been suggested that a dysfunction of the TRN may underlie schizophrenia-associated behavioral phenotypes. In line with the developmental hypothesis of schizophrenia, exposure to environmental risk factors during critical periods of neurodevelopment, combined with genetic predisposition, increases the risk of developing the disorder. Consequently, various “multiple-hit” models have been described to mimic the progression of the disease. In a double-hit mouse model for schizophrenia (involving neonatal exposure to an N-Methyl-D-aspartic acid receptor antagonist followed by post-weaning social isolation), impairments in specific subpopulations of GABAergic neurons were observed in adult animals (Percelay et al., 2024). As previously mentioned, TRN neurons abundantly express PV. However, PV-positive neurons are not exclusive to this nucleus, as they are widely distributed across both cortical and subcortical regions of the mammalian brain. A hallmark of these neurons is their fast-spiking activity, driven by calcium dynamics regulated by PV (Rupert and Shea, 2022). However, their high metabolic demand renders them particularly vulnerable to oxidative and nitrosative stress (Steullet et al., 2018). Furthermore, PV-positive interneurons are structurally associated with specific extracellular matrix components known as PNNs. Beyond their established role in synaptic transmission, as described in the tetrapartite synaptic model (Chelini et al., 2018; Rupert and Shea, 2022), PNNs are essential for antioxidant defense mechanisms (Cabungcal et al., 2013). In their double-hit schizophrenia related model, Klimczak et al. (2024) reported a reduction in the proportion of PV-positive cells associated with PNNs in the TRN and a reduction of the area covered by PV-positive puncta in the MDT. These alterations in PV-positive cells were accompanied by a decreased expression of the N-Methyl-D-aspartic acid receptor subunit GluN1 and of PSA-NCAM in the TRN. While this study did not evaluate whether pharmacological interventions could reverse or mitigate TRN impairments, other models have addressed this question. One such model involves neurodevelopmental disruption caused by prenatal exposure to the mitotoxic compound methylazoxymethanol acetate (MAM), commonly referred to as the MAM model. Rats exposed to MAM *in utero* exhibited impaired TRN function, characterized by the loss of PV-positive interneurons and reduced PNN density (Zhu et al., 2021). Interestingly, the same study demonstrated that juvenile treatment with N-acetylcysteine, a compound with antioxidant properties, prevented TRN impairments in adult MAM-exposed rats. Similarly, juvenile N-acetylcysteine treatment has been shown to preserve PV-positive interneurons and PNNs in the PFC of adult rats subjected to NVHL (Cabungcal et al., 2014).

In schizophrenia, most proposed biomarkers for identifying neurobiological changes are related to EEG activity (Barros et al., 2021). The role of the TRN in EEG activity has been clearly demonstrated using direct optogenetic manipulation of PV cells in mice, revealing that dysfunctional TRN-PV neuron activity may contribute to schizophrenia-related abnormalities in cortical delta, gamma, and spindle rhythms (Thankachan et al., 2019). Additionally, a reduction in sleep spindle activity involving the TRN-MDT-PFC loop has been described in the disease (Ferrarelli and Tononi, 2017). TRN dysfunction in patients with schizophrenia directly influences key neurophysiological changes reflected in oscillatory activity (Celada et al., 2013). Interestingly, rats with NVHL exhibit features of EEG hypofrontality (reduced activity in the frontal regions, evidenced by decreased power in specific frequency bands; Ahnaou et al., 2007; Valdés-Cruz et al., 2012; **Figure 2B**), similar to what is observed in patients with schizophrenia (Knyazeva et al., 2008). Specifically, a global reduction in absolute power at alpha (8–12.5 Hz) and beta (13–30 Hz) frequencies, as well as an increase in relative alpha power in the prefrontal cortex, has been observed in patients (Knyazeva et al., 2008). These findings may correspond to the reduction in the 1–30 Hz absolute power bandwidth across prefrontal, parietal, and temporal cortices in the rats with NVHL (Ahnaou et al., 2007; Valdés-Cruz et al., 2012). This impaired cortical EEG activity highlights a clinical comorbidity of psychosis and epilepsy. This comorbidity is more commonly observed in patients with partial seizures, while co-occurrence with absence epilepsy is rare, likely due to differences in the typical age of onset for psychosis and epilepsy (Adachi et al., 2011; Adachi and Ito, 2022). In the NVHL model, interictal desynchronization (Neymotin et al., 2010) and spike-wave discharge activity have been documented (Lee et al., 2014; Magdaleno-Madrigal et al., 2017). This aberrant oscillatory activity involves the cortico-thalamo-hippocampal circuit, including the PFC, TRN, and hippocampal CA1, among other regions (Wang et al., 2009; Han et al., 2012). Notably, in rats with NVHL, high-frequency electrical stimulation of the rostral TRN (DBS-TRN) was found to reduce the duration, frequency, and amplitude of spike-wave discharge activity. This suggests that DBS-TRN may modulate the PFC-MDT-hippocampus circuit (Magdaleno-Madrigal et al., 2017) and represents a promising non-pharmacological therapeutic approach for the treatment of schizophrenia (Gerardo and Manuel, 2020).

In summary, the TRN receives multiple direct and indirect inputs from regions affected in NVHL, such as the PFC (**Figure 1**), and given its role in this complex cortico-limbic-thalamic circuit, the neuroplasticity alterations reported here may result from indirect effects of early-life hippocampus-PFC disconnection, potentially driven by changes in mesolimbic dopaminergic tone triggered by NVHL, as NAcc hyperactivity leads to sustained inhibition of the VP, which normally restrains MDT spontaneous activity, ultimately causing PFC disinhibition, where NVHL also induces PV-positive interneuron loss (Cabungcal et al., 2014) and dysfunction (Tseng et al., 2008), and these mechanisms may indirectly affect TRN plasticity, since the PFC is a central hub of the cortico-limbic circuit and also projects to the TRN, potentially directly regulating the plasticity of its neurons (**Figure 1**).

## Thalamic Reticular Nucleus Differences between Humans and Rodents, Limitations of the Neonatal Ventral Hippocampus-lesioned Model Findings for the Schizophrenia Pathophysiology

Rodents and humans differ in the morphology, cellular composition, and function of their TRN. In both species, the thalamus is encased in a GABAergic shell, but the human TRN is proportionally smaller and more intricately organized, reflecting the larger human brain (Pritz, 2025). Compared with rats, human neocortical neurons that interface with the TRN show lower membrane capacitance and distinct threshold potentials, influencing TRN network dynamics. While the rodent TRN is well characterized for roles in thalamocortical oscillations, sensory gating, and sleep spindle regulation, the human TRN likely supports more complex sensory processing and higher-order cognitive integration, as indicated by imaging evidence of its unique modulation of thalamic relay nuclei and interhemispheric communication (Viviano and Schneider, 2015; Pritz, 2025).

Thus, the dendritic spine degeneration observed in TRN neurons of rats with NVHL may be linked to the impaired sensory gating and heightened exploratory activity reported in these animals, which could reflect certain positive-like symptoms of schizophrenia. Nevertheless, because the TRN also participates in higher cognitive processes, findings on TRN plasticity should be interpreted cautiously when considering their relevance to schizophrenia pathophysiology. Even so, the observation that early-life hippocampal–prefrontal disconnection in rats can induce neuroanatomical changes in the TRN supports the neurodevelopmental hypothesis of schizophrenia, on which the NVHL model is based (Lipska and Weinberger, 2002) and which aligns with the mechanistic approach presented in this work (**Figure 1**).

## Thalamic Reticular Nucleus Dysfunction in Other Psychiatric Diseases

TRN dysfunction is not exclusively associated with schizophrenia, at least at the preclinical level. For example, peripubertal stress in mice induces a depression-related behavioral phenotype and TRN dysfunction in adulthood (Alcaide et al., 2024). This dysfunction is primarily characterized by a reduction in PV-positive interneuron connectivity and plasticity-regulating molecules, such as PSA-NCAM and specific N-Methyl-D-aspartic acid receptor subunits, and is accompanied by a decrease in TRN volume. These structural and molecular alterations in the TRN suggest that its prolonged developmental trajectory renders it particularly vulnerable to early-life adversity. This vulnerability may contribute to the pathophysiology of mental health disorders, particularly in females, where stress exerts more pronounced effects on the TRN, including reductions in nucleus volume (Alcaide et al., 2024).

Although limited research has focused on the TRN in depression, when combined with findings from schizophrenia-related models, the TRN may represent a common neurobiological substrate for some behavioral phenotypes. Restoring TRN function by mitigating oxidative/nitrosative stress or by DBS-TRN-related strategies could thus serve as a potential therapeutic contributor to neurodevelopmental and stress-related disorders. However, further studies, particularly at the clinical level, will be needed to test this hypothesis in the coming years.

## Conclusions and Perspectives

In conclusion, substantial evidence suggests that deficits in TRN functionality may contribute to the pathophysiology of schizophrenia, based on the assumption that PFC hypofunction could reduce TRN stimulation. This reduction may, in turn, weaken the inhibitory effect of TRN, which is critical for controlling thalamic relay nuclei. In schizophrenia, the most adversely affected relay nucleus is likely the MDT. These findings align with the hypothesis proposed by our group and others, emphasizing oxidative stress in the brain as a core mechanism in schizophrenia pathophysiology. Mitigating or controlling oxidative stress represents a promising prospect for the development of safer and more effective pharmacological interventions, moving beyond the traditional modulation of monoaminergic systems in the brain (Leza et al., 2015; Steullet et al., 2018; Tendilla-Beltran and Flores, 2020). Despite the challenges posed by its complexity, we anticipate that future research on the thalamus will increasingly highlight the role of TRN in schizophrenia.

## Data Availability

*Not applicable.*
